# Program esilence 1.0 - self-regulation program in food education via instagram-loricorps, study protocol

**DOI:** 10.1192/j.eurpsy.2021.315

**Published:** 2021-08-13

**Authors:** V. Lemieux, J. Monthuy-Blanc, N. Moreau

**Affiliations:** 1 Biomedical Sciences, Université du Québec à Trois-Rivières, Trois-Rivières, Canada; 2 Education, Université du Québec à Trois-Rivières, Trois-Rivières, Canada; 3 Service Social, Université d’ottawa, Ottawa, Canada

**Keywords:** Physical self-perceptions, New technologies, ehealth, Dysfunctional eating attitudes and behaviours

## Abstract

**Introduction:**

Social medias are seen as a risk factor for mental health because they increase body dissatisfaction and decrease self-esteem. This program is based on alimentation and physical well-being by relying on integrated intuitive eating and physical self-esteem. This program, implemented in a community setting use social media (i.e. Instagram-Loricorps), is composed of 12 monthly 180-second video capsule that address themes related to the promotion of body sensations and intuitive movement.

**Objectives:**

The main objective of this study is to evaluate the effects of the program into the physical environment targeting the physical self-perceptions (PSP). Specifically, this study evaluates whether the eSILENCE 1.0 Program improves the level of PSP related to nutrition and explores the changes in the level and variability of the PSP.

**Methods:**

This project is a mixed sequential explanatory study. 300 participants (Experimental Group [EG; N=200], Control Group [CG; N=100]) are targeted. Online nomothetic questionnaires evaluate occupational changes and PSP in relation to alimentation and are completed by the EG and the CG at pre-test, mid-test and post-test. Online idiographic questionnaires assess PSP and are completed by the EG before and after each video capsule and by the CG once a month without viewing the capsules. Following a preliminary analysis, a focus group will be formed to explain and deepen these results. Participants (N=5) will be recruited voluntarily into the EG.

**Results:**

to come.

**Conclusions:**

Analysis of quantitative data will be used to assess the effectiveness of the program and analysis of qualitative data will provide an in-depth understanding of the linkages between the variables.

**Disclosure:**

No significant relationships.

